# Rational Design of Electric Field-Responsive Building Blocks for All-Organic 2D Magnetoelectric Materials

**DOI:** 10.1021/jacs.5c02910

**Published:** 2025-06-17

**Authors:** Kílian Jutglar-Lozano, Mercè Deumal, Jordi Ribas-Arino, Stefan T. Bromley

**Affiliations:** 1 Departament de Ciència de Materials i Química Física & Institut de Química Teòrica i Computacional (IQTCUB), 16724 Universitat de Barcelona, c/Martí i Franquès 1-11, Barcelona 08028, Spain; 2 Institució Catalana de Recerca i Estudis Avançats (ICREA), Passeig Lluís Companys 23, Barcelona 08010, Spain

## Abstract

Development of technologically promising magnetoelectric materials, where magnetic properties can be controlled by electric fields (E-fields), has focused on inorganic systems. Here, we propose a strategy for modulating magnetic exchange coupling (*J*) in purely organic systems through experimentally realizable E-fields. Our approach leverages two established concepts: (i) E-field-induced twisting of dipolar organic linkers and (ii) control of *J* via conformational changes in organic diradicals. Using density functional theory calculations, we investigated the effects of applied E-fields on diradicals with two coplanar spin-carrying trioxotriangulene (TOT) radicals connected by dipolar aryl linkers. We find that E-fields induce significant conformational changes in the linkers (twisting) that alters π-conjugation and, in turn, the magnetic *J* coupling between TOT radicals. In-plane E-fields twist the linkers toward the plane of the radicals, enhancing π-conjugation and increasing AFM coupling. Out-of-plane E-fields induce more orthogonal linker conformations and decrease the coupling strength. The magnetoelectric response depends on a combination of steric hindrance, π-conjugation, and polarization. Significant and measurable cumulative changes in *J* of up to 3.9 meV could be achieved by using in-plane and out-of-plane E-fields of up to 0.5 V/Å. In some cases, applied E-fields can also induce switching between paramagnetism and antiferromagnetism. Calculations on a 2D covalent organic framework (COF) based on a network of TOT radicals and dipolar linkers confirm that this approach is also viable for extended systems. Such COFS could also display E-field induced ferroelectric responses. Overall, our proof-of-principle study highlights the interplay between molecular structure, E-fields, and magnetism and establishes an innovative and chemically rational framework for developing all-organic magnetoelectric materials.

## Graphical abstract

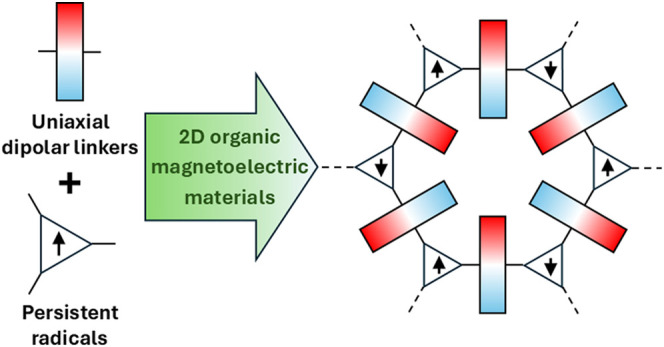

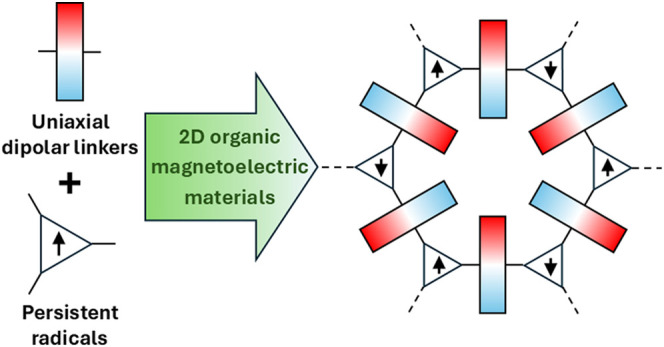

## Introduction

The development of magnetoelectric materials in which magnetic and electrical properties are coupled is opening up a wide range of new opportunities for technological applications.^1–3^ The control of magnetic properties by applied electrical fields (E-fields) is often referred to as the converse magnetoelectric effect (CME). The CME avoids the need for indirect and inefficient generation of magnetic fields by electric currents^4^ and thus opens the door to new future applications that are inherently compatible with existing E-field gated electronics (e.g., low power spintronic devices, high-density magnetic data storage).^5,6^ Several groundbreaking experimental and theoretical studies have reported CME-based phenomena in a wide range of transition-metal-based materials and molecular systems. Examples of results achieved so far include electrical control of (i) magnetic order in inorganic materials,^7–11^ (ii) spin-crossover in nanoparticles,^12^ (iii) spin-direction or spin-spectrum in individual ions,^13,14^ (iv) spin coherence in molecular nanomagnets,^15^ and (v) spin states via spin chirality.^16,17^ The E-field-based modulation of spin exchange couplings (*J*) has also been achieved or predicted in few selected transition metal-containing molecular systems.^18–23^ While many organic magnetic systems have been reported, examples of fully organic extended CME materials remain scarce.^24^ Organic CME materials would have several advantages over the use of inorganic materials (e.g., structural flexibility, low spin–orbit coupling, ease of functionalization, low energy synthesis, sustainability) and could thus potentially serve as a new platform for highly versatile spintronic device technologies. One of the few reported examples of an all-organic CME system is based on donor–acceptor charge transfer in a disordered material based on carbon nanotubes and fullerenes.^25^ In organic molecular junctions, two examples of electrical control of magnetism include (i) modest changes of *J* in a triradical via a gate voltage^26^ and (ii) spin-state tuning in a diradical via a bias voltage across the junction.^27^ In this work, we report a new approach for exploiting molecular-scale magnetoelectric responses to design all-organic CME materials. We highlight our approach by rational design of synthetically viable diradical building blocks in which continuous modulation of *J* is achieved via E-field-induced conformational changes of dipolar linkers.

Our strategy builds upon two experimentally established concepts: (i) E-field-induced conformational twisting of dipolar organic axial linkers and (ii) control of *J* via mechanical or thermally driven conformational changes in organic diradicals. The former effect has been reported for organic materials^28,29^ and single molecules on surfaces.^30^ With respect to the latter, conformational changes in open-shell molecular dimers induced by mechanical^31–33^ or thermal^34^ means have been shown to allow tuning of *J*. We have demonstrated that this conformational response can also be induced in extended materials, whereby mechanical strain and stress can be used to control magnetic interactions in organic radical-based 2D covalent organic frameworks (2D-COFs).^35–37^

Herein, we consider systems in which stable organic spin centers are axially linked by dipolar functionalized aryl rings. The dipolar linkers can act as twistable E-field-actuated conformational molecular switches, which can modulate the degree of π-conjugation between the radical spin centers.^38^ Using accurate electronic structure calculations, we explore whether such an approach can lead to measurable changes in *J* between the unpaired spins upon application of experimentally realizable E-field strengths.

In this proof-of-principle study, we employ trioxotriangulene (TOT) radicals to provide the unpaired spin centers (see Figure 1). TOT is a stable nanographene-like organic radical that has been used as a building block in a range of open-shell materials.^39–41^ Among the numerous known persistent organic radicals, TOT has a relatively unique set of features for our study: (i) a planar nondipolar structure that leads to a delocalization of the unpaired electron, which can promote strong magnetic couplings, (ii) a 3-fold symmetric structure that allows it to be used as a trivalent node in 2D-COFs (e.g., when combined with uniaxial linkers), and (iii) a relatively high chemical stability with respect to other competing radicals that satisfy (i) and (ii). We note that point (i) is also supported by a theoretical comparison of a set of open-shell nanographenes connected by nondipolar conjugated linkers, which predicted that TOT-based systems provide relatively high antiferromagnetic (AFM) couplings.^32^ As an essential ingredient in all our studied systems, the TOT centers are axially connected by conjugated dipolar linkers. For promoting E-field responsiveness, we consider linkers, which adhere to the following criteria: (i) allow for a uniaxial linkage between two TOT radicals, (ii) have a permanent dipole, and (iii) allow for π-conjugation with TOT. Specifically, we consider four such linkers: difluorobenzene (DFB), difluoronaphthalene (DFN), difluoroanthracene (DFA), and aminonitroanthracene (ANA) (see Figure 1). The series of three linkers (DFB, DFN, and DFA) were chosen to maintain the same dipole-inducing substitution (i.e., two F atoms) while systematically increasing the number of fused rings in the linkers. This variation was primarily selected to vary the degree of steric hindrance between the linker and the TOT radicals. We note that difluoro substitution has also been used in experimental studies to create E-field-responsive dipolar twistable linkers.^29,42^ The ANA linker allows us to explore the impact of a stronger permanent dipole while maintaining the same degree of steric hindrance as that of the DFA linker. The dipole of ANA arises from the electron-donating character of the amino group and the electron-withdrawing character of the nitro group. The position of the NO_2_ and NH_2_ substituents in ANA mirrors the arrangement of these substituents in 2,5-di­(ethynyladamantanyl)-4-(dimethylamino)­nitrobenzene that has been shown to allow for controlled single-molecule rotation on a surface using the E-field of a scanning tunneling microscope.^30^ In addition to these dipolar linkers, we also include a nondipolar unsubstituted phenyl ring linker (Ph) for comparison. We also note that these five linkers and the TOT radical are all stable, readily synthesizable organic building blocks. The combined systems of coupled TOT radicals allow external E-fields to tune the dihedral twist angle of the linkers. In turn, the resulting change in π-conjugation has the potential to induce changes in the *J* between TOT spin centers. This principle can be applied to both molecular diradicals and extended covalently linked materials (e.g., COFs or conjugated polymers) (see the right-hand side of Figure 1). We note that in some conformations of the linkers, the coupling between the radical centers can become negligible and thus the diradicals formally become biradicals. For consistency, we refer to our molecular systems as diradicals throughout.

**Figure 1:**
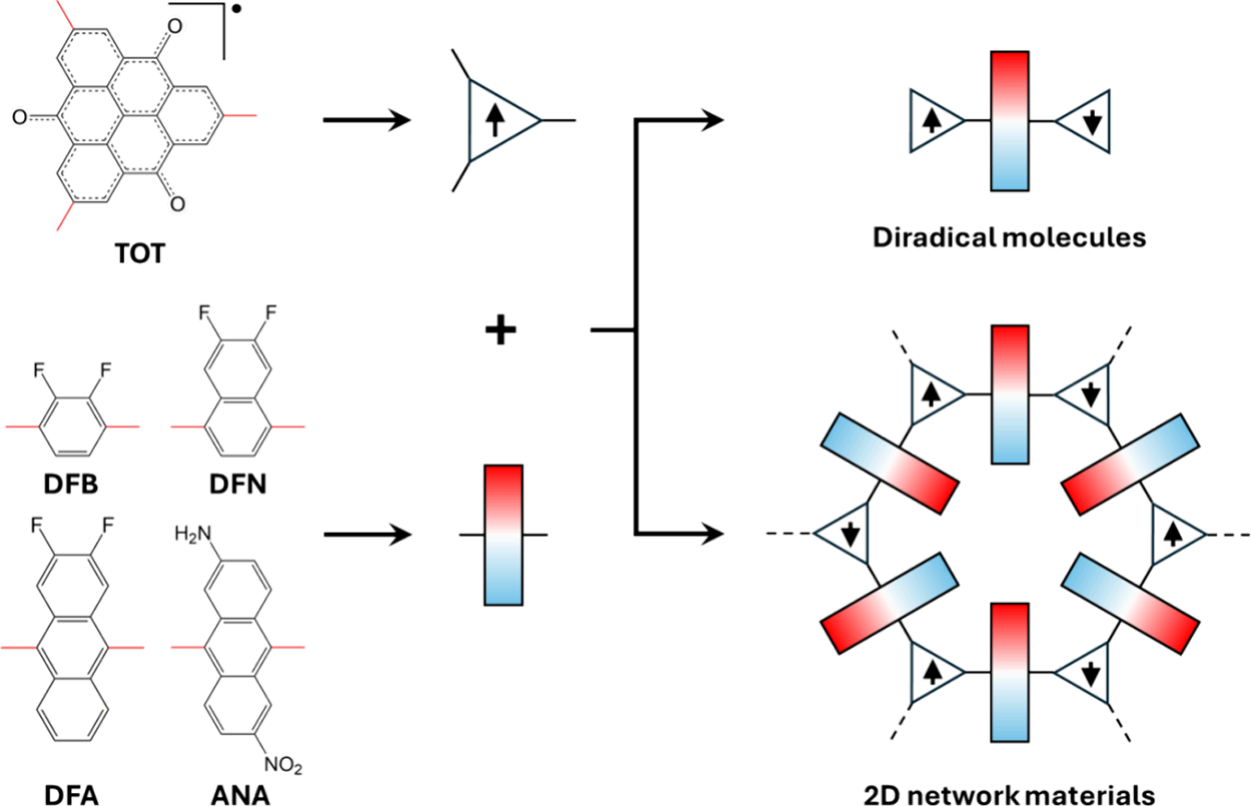
Scheme showing how TOT nanographene radicals and conjugated dipolar linkers (difluorobenzene, DFB; difluoronaphthalene, DFN; difluoroanthracene, DFA; and aminonitroanthracene, ANA) can be combined to form diradical molecules and 2D network materials. Linkage sites for combining molecular building units are highlighted in red in the chemical structures.

Specifically, we assess the magnetic responsiveness of such conformationally tunable systems with respect to (i) the dipolar character of the linkers, (ii) the ease of linker rotation, and (iii) the strength and direction of the applied E-field. We systematically study these factors for a set of diradical molecular systems and assess the implications for the development of fully organic 2D CME materials.

## Methods

Relaxed structures of all molecular diradicals were obtained via density functional theory (DFT)-based optimizations using the PBE0 exchange-correlation functional,^43^ and a 6-311g­(d,p) basis set, as implemented in the Gaussian09 ^44^ code. We note that DFT calculations using the PBE0 functional have been found to provide an accurate description of magnetic coupling in other systems of covalently linked organic radicals as compared to experiment^36,45^ and high-level quantum chemical calculations.^46^ We have also confirmed that the results from PBE0 calculations for our TOT–linker–TOT systems with dipolar conjugated linkers are quantitatively comparable to those from CASSCF­(12,12)/NEVPT2 wave function-based calculations carried out with the ORCA code^47^ (see the Supporting Information (SI)). We have also confirmed that the 6-311g­(d,p) basis set gives very comparable results to calculations using a larger def2-TZVPP basis set (see the SI). Dispersion interactions were included using Grimme’s D3 scheme in all cases.^48,49^

All optimizations were carried out in the open-shell singlet ground state. It is expected that 2D materials of covalently bonded nanographene units should maintain the coplanarity of the TOT centers.^50^ As such, appropriate geometrical constraints were applied to maintain coplanarity in the molecular systems to mirror their role as models for extended 2D systems (see the SI). All optimizations employed tight convergence settings.

Uniform E-fields were applied to all of these molecular systems in both in-plane and out-of-plane directions with respect to the plane of the TOT units. Applied E-fields were included via the “Field” keyword in the Gaussian code, which allows the user to define the direction and strength of the field. In such calculations, a new term accounting for the potential due to the uniform E-field is added to the external potential of the Kohn–Sham Hamiltonian. The Kohn–Sham equations are then solved by taking into account the effective potential. Accordingly, the relaxation of the electronic density is self-consistently evaluated with the influence of the applied E-field. For each diradical system, we considered a range of E-field strengths between 0 and 0.5 V/Å in steps of 0.025 V/Å. We note that such E-field strengths are readily achievable with devices based on dual ionic liquid gating that can generate up to 0.4 V/Å,^51^ or via STM tips that can generate E-fields up to 2.0 V/Å.^52^ Conformational energy profiles as a function of the E-field were carried out by means of constrained optimizations in which the value of the dihedral twist angle between the linker and the TOT radicals (θ) was fixed at a series of values from 15 to 165° in steps of 15°. Conformations for θ < 15° were difficult to converge due to the high steric hindrance between the linkers and TOT radicals. This indicates that these conformations would not be easily experimentally realizable and were excluded from our study.

Magnetic exchange spin couplings (*J*) between the unpaired spins on the TOT radicals were determined using the Heisenberg–Dirac–Van Vleck Hamiltonian *Ĥ* = −2*JŜ*_1_ *Ŝ*_2_, where *J* is computed from the energy difference between the open-shell singlet and triplet states Δ*E* ^S–T^ = 2*J*. Note that within a DFT framework, the open-shell singlet is obtained using the broken symmetry (BS) approach, resulting in Δ*E* ^S–T^ = 2­(*E*_BS_^S^ – *E* ^T^)/(1 + *S*_ab_), where *S*_ab_ is the overlap integral between the magnetic α and β singly occupied molecular orbitals of the BS solution. In this case, there is minimal overlap between the two singly occupied molecular orbitals, which are mainly localized on the TOT radicals (*S*_ab_ ≈ 0), and thus *J* = *E*_BS_^S^ – *E* ^T^ (i.e., the Noodleman projection). *J* values computed using the Yamaguchi formula were found to be virtually identical to the Noodleman-derived values (see the SI). All reported *J* values correspond to vertical differences between the triplet and single states at the optimized geometry of the respective singlet. The singlet states were optimized without applying any spin contamination corrections. Using Noodleman’s projection scheme, as implemented in the ORCA code, we verified that spin contamination did not significantly affect geometries or calculated *J* values. Specifically, for the DFB system at zero field, the root mean squared difference between the projected and unprojected geometries for the BS solution is 0.0003 Å. In addition, the difference between projected and unprojected *J* is extremely small (0.003 cm^–1^). We also note that *J* values obtained from adiabatic energy differences between singlet and triplet states were found to be negligibly different from the vertical *J* values (see the SI).

Periodic DFT calculations of a hexagonally ordered 2D material based on TOT radicals connected by ANA linkers were performed using the all-electron FHI-AIMS code.^53^ The general structure of such a material consists of a covalently linked honeycomb array of trivalent spin centers bridged by uniaxial dipolar linkers (see Figure 1). A 40 Å vacuum in the out-of-plane *z*-direction was employed to avoid interlayer interactions. Both the AFM and ferromagnetic (FM) solutions were described by using the PBE0 hybrid functional. The FM solution was computed by using the relaxed structure of the AFM ground state. A 6 × 6 × 1 Monkhorst–Pack grid was used for the **k**-point sampling. All periodic calculations employed a “light” numerical atom-centered orbital basis set,^54^ which is similar in accuracy to the polarized triple-ζ Gaussian basis set used for the molecular systems.^55^ Dispersion interactions were included using the Tkatchenko–Scheffler method.^56^ Atomic positions and cell parameters were optimized in the AFM solution until a maximum force of 10^–3^ eV/Å was achieved. Subsequently, a single-point calculation of the FM solution was performed to evaluate *J* between the unpaired electrons of the radical subunits. The θ-dependency of *J* used a synchronous rotation of all linkers in steps of 15° of θ, following the procedure used in the molecular diradical calculations. Conformationally constrained systems were obtained by freezing the *z* Cartesian component of key linker atoms to prevent rotation (see the SI). The relative energies of the 2D material were normalized with respect to the number of linkers in the cell for comparison with the results for the diradical molecular systems.

## Results and Discussion

In the following, we first focus on diradical molecular systems, where we present a comparative analysis of the relationship between *J* and linker conformation and how both these factors are affected by applied E-fields of varying strength and direction. Subsequently, we compare the E-field response of the ANA-based diradical with that of the analogous open-shell 2D network material.

### Dependence of *J* on the Linker Twist Angle

We first consider the magnetic coupling interaction, *J*, as a function of θ. We define θ = 0° for the case when the linker and radicals are in the same plane. Consequently, θ = 90° describes the situation when the linker is orthogonal to the plane of the radicals. In Figure 2a, we show the two limiting cases of θ = 0° and θ = 90° for the case of ANA linked to two TOT radicals. Due to symmetry, any conformation between 90 and 360° can be mapped onto a given conformation within the 0 to 90° range.

**Figure 2:**
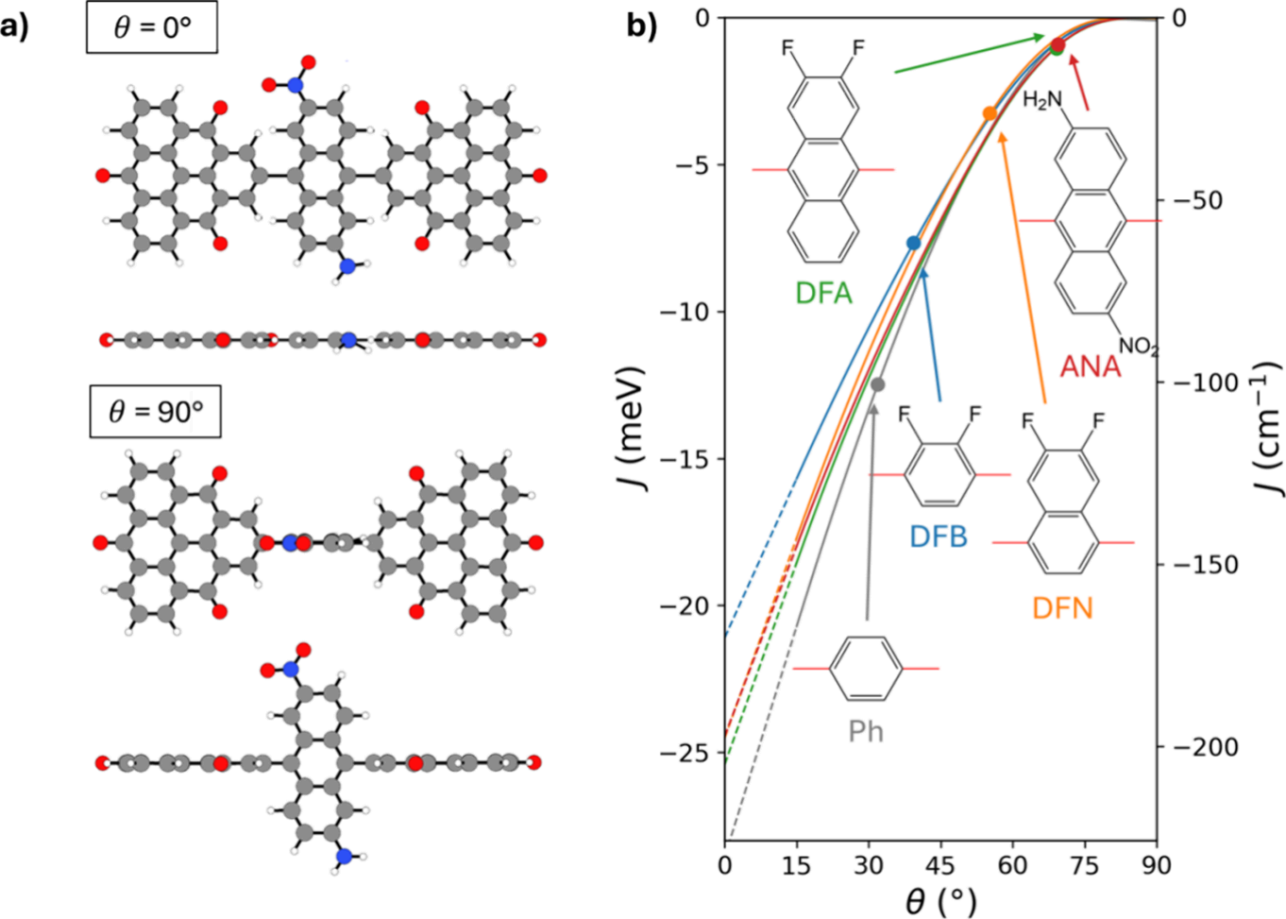
(a) Top and side views of the ANA-linked diradical for θ = 0° and θ = 90°. Atom color key: C—gray, O—red, N—blue, H—white. (b) Variation of *J* as a function of θ for the five considered linkers: Ph (gray), DFB (blue), DFN (orange), DFA (green), and ANA (red). For each linker, the value of θ in their respective relaxed diradical structure is indicated with a solid data point. Bold lines indicate regions for explicitly calculated data (guide to the eye through discretely sampled data), while dashed lines for θ < 15° indicate extrapolations.

In Figure 2b, we show how *J* varies with respect to changes of the twist angle of each dipolar linker for the range θ = 0–90°. In all cases, we find a negligible coupling for θ = 90° and a monotonic increase in AFM coupling (i.e., more negative *J* values) when decreasing θ. This implies that the systems would be paramagnetic when the linkers are orthogonal and would become progressively more strongly coupled as the linkers twist toward the plane of the radicals. This general trend is in line with the fact that lower θ values (i.e., higher planarity) lead to higher magnetic coupling due to higher linker-radical π-orbital overlap, and vice versa. We also observe notable linker-dependent differences in *J* for smaller θ values. For example, comparing *J* values for each difluoro-based linker at θ = 15°, we see that DFB has the smallest AFM coupling (−15.7 meV/–127 cm^–1^), followed by DFN (−17.7 meV/–143 cm^–1^) and then DFA (−18.5 meV/–150 cm^–1^). We can see that the tendency to increase the AFM coupling magnitude in this regular series follows a corresponding increase in the degree of conjugation of the linkers. We note that our general approach is not limited to systems with AFM coupling. For example, the use of antiaromatic dipolar linkers would be expected to provide a basis for all-organic FM-coupled magnetoelectric systems.^57^

The size and structure of each diradical also leads to their equilibrium relaxed geometries having a range of favored twist angles (θ_0_). This variation is mainly due to the differing degrees of steric hindrance between the linkers and the radicals. As a reference, the nondipolar single Ph linker has a relatively low steric hindrance with the TOT radicals, allowing a rather planar relaxed conformation (θ_0_ = 32°) with *J*_0_ = −12.5 meV (see also ref 32). DFB is the smallest dipolar linker for which significant steric hindrance is only encountered for relatively small values of θ. As such, DFB is only mildly perturbed with respect to the Ph linker. The resulting slightly increased out-of-plane twist angle when relaxed (θ_0_ = 39°) leads to a relatively smaller AFM coupling strength of *J*_0_ = −7.7 meV. For progressively larger linkers (see Figure 2b) with two fused aromatic rings (DFN) or three fused rings (DFA and ANA), the steric hindrance increases more significantly. This is due to the corresponding increase in nonbonded interactions between the terminal hydrogen atoms on the extra rings and the TOT radicals (see Figure 2a). Following this expected tendency, we see that DFN has an intermediate θ_0_ value of 55° with a corresponding *J*_0_ = −3.2 meV. For DFA and ANA, the higher steric hindrance leads to the same higher θ_0_ value (69°), leading to relatively low relaxed AFM coupling strengths (*J*_0_ = −1.0 and −0.9 meV, respectively).

### Tuning θ and *J* with Applied E-Fields

Here, we examine how applied E-fields interact with our dipolar linkers to induce changes in θ and thus the corresponding changes in *J*. We consider the application of E-fields in two distinct directions: (i) in-plane and (ii) out-of-plane with respect to the common plane of the TOT radicals (see Figure 3a). The in-plane E-field direction favors smaller values of θ, while an out-of-plane E-field tends to increase θ. To compare the E-field-induced conformational response of each linker, we refer to the change in θ relative to that of the initial relaxed conformation (Δθ_0_ = θ – θ_0_). In Figure 3b, we show how Δθ_0_ varies with respect to applied in-plane and out-of-plane E-fields varying in magnitude between 0 and 0.5 V/Å in each direction. Generally, a higher E-field magnitude leads to a larger Δθ_0_ response for each linker. For in-plane E-fields, the maximal response would be to achieve θ = 0°, whereas θ = 90° is the limiting conformational response for out-of-plane E-fields.

**Figure 3:**
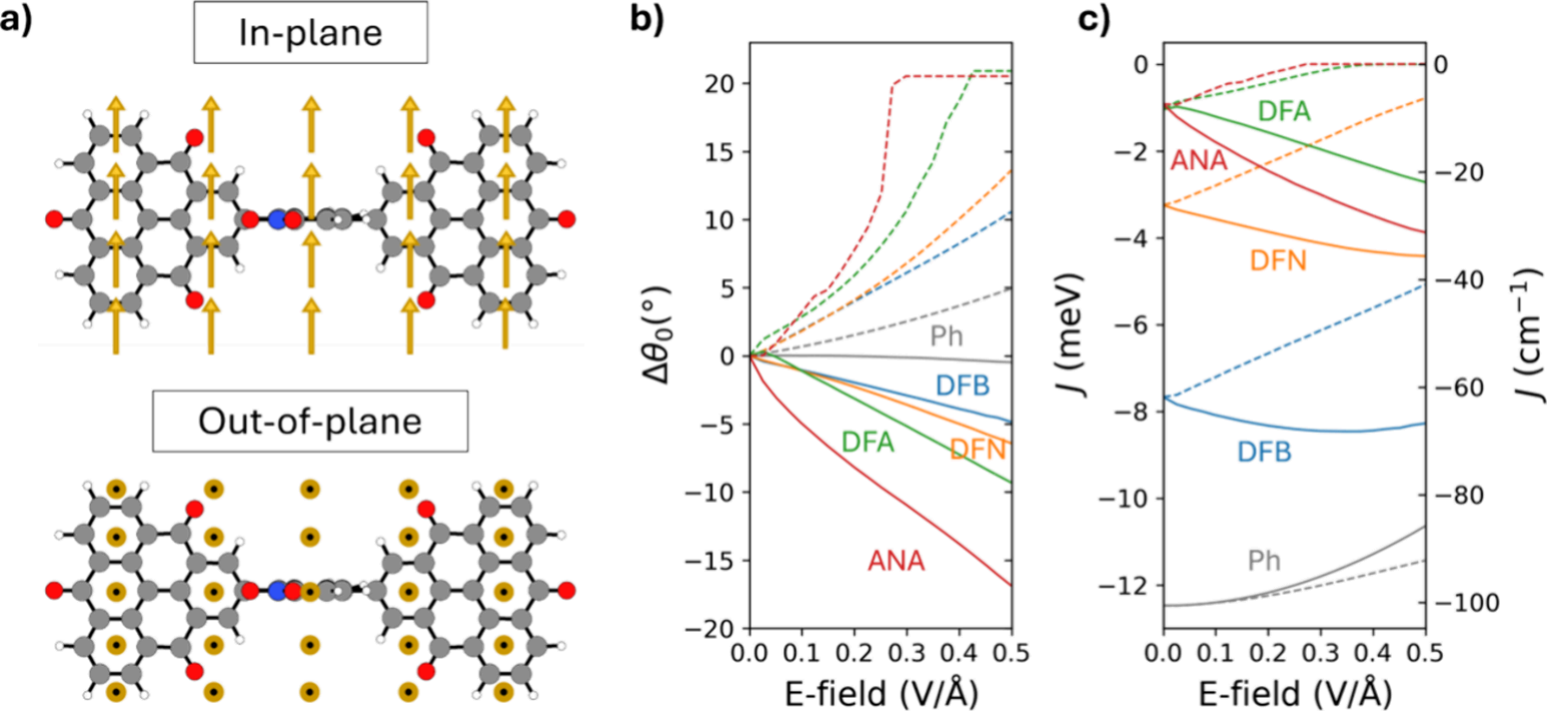
(a) Direction of applied E-field indicated by yellow arrows. Variation of (b) dihedral angle of each linker relative to the zero-field relaxed conformation (Δθ_0_ = θ – θ_0_) and (c) AFM coupling strength (*J*) with respect to applied E-field strength. In-plane and out-of-plane E-fields are indicated by solid lines and dashed lines, respectively. The E-field strength in each case ranges between 0 and 0.5 V/Å in steps of 0.025 V/Å.

The degree of the Δθ_0_ response with respect to the strength of the applied E-field varies significantly for each linker. Generally, the smaller linkers, DFB and DFN, have a conformational responsiveness that is similarly lower than that of the larger linkers, DFA and ANA. In all cases, the rate of change of Δθ_0_ for small E-fields is larger for out-of-plane fields than for in-plane fields. For ANA and DFA, a near-orthogonal conformational limit is reached with a Δθ_0_ of approximately +20° for out-of-plane E-field strengths of 0.3 and 0.425 V/Å, respectively, where the E-field response saturates (see Figure 3b and Table 1). For DFB and DFN, a relatively high out-of-plane E-field of 0.5 V/Å can give rise to similar Δθ_0_ values of +11 and +14° (see Figure 3b and Table 1). However, in these cases, the relatively lower values of θ_0_ mean that the linkers are still far from the orthogonal limit. With respect to the dipolar linkers, the Δθ_0_ out-of-plane conformational response at 0.5 V/Å is significantly reduced to +5° for the reference nondipolar Ph linker.

**Table 1:** Summary of the Maximum Conformational and Magnetic *E*-Field Responses of Each Considered Linker with Respect to the Corresponding Relaxed System with No Applied E-Field^c^

	relaxed		in-plane *E*-field		out-of-plane *E*-field		total response	
	**θ**_**0**_ ^a^	***J*_0_** (meV/cm^–1^)^b^	**Δθ_0_** ^a^	**Δ*J*_0_** ^b^	**Δθ_0_** ^a^	**Δ*J*_0_** ^b^	**Δθ_tot_** ^a^	**Δ*J*_tot_** ^b^
**Ph**	32	–12.5/–101	–1	+1.9/+15	+5	+1.1/+9	6	0.8/6
**DFB**	39	–7.7/–62	–5	–0.6/–5	+11	+2.6/+21	16	3.2/26
**DFN**	55	–3.2/–26	–6	–1.2/–10	+14	+2.4/+20	20	3.6/30
**DFA**	69	–1.0/–8	–9	–1.7/–14	+20	+1.0/+8	29	2.7/22
**ANA**	69	–0.9/–7	–16	–3.0/–24	+20	+0.9/+7	36	3.9/31

a All dihedral twist angles θ are given in degrees (°).

b All magnetic couplings *J* are given in meV and cm^–1^.

c Columns 2 and 3 show the relaxed values of θ (θ_0_) and *J* (*J*_0_) for each linker. Columns 4 (6) and 5 (7) show the maximum changes in θ (Δθ_0_) and *J* (Δ*J*_0_) with respect the respective relaxed values upon application of a 0.5 V/Å in-plane (out-of-plane) E-field. Columns 8 and 9 show the cumulative absolute differences in θ (Δθ_tot_) and *J* (Δ*J*_tot_) (i.e., |value­(out-of-plane) – value­(in-plane)|) when both applied E-field directions are used.

For in-plane E-fields, the planar limit is not reached for any linker. As for applied out-of-plane E-fields, DFN and DFB have a similar relatively low responsiveness to in-plane E-fields, followed by DFA and then ANA in order of increasing responsiveness. The highest magnitude of the observed Δθ_0_ values for the largest considered in-plane E-field varies from −5° for DFB to −16° for ANA (see Figure 3b and Table 1). For the nondipolar Ph linker, the corresponding in-plane Δθ_0_ value is found to be only −1°.

In Figure 3c, we show how the E-field-induced conformational changes lead to corresponding changes in *J*. Out-of-plane E-fields increase Δθ_0_, which decreases π-orbital overlap and lowers the AFM coupling strength (i.e., leads to smaller *J* values in absolute value). In the extreme cases, when the strength of the applied out-of-plane E-field causes the ANA and DFA linkers to become almost perfectly orthogonal to the TOT linkers (see Figure 3b), *J* becomes negligible (see Figure 3c). In these cases, as the relaxed systems have relatively large θ_0_ values with correspondingly small low AFM couplings, the change in *J* upon going from the relaxed systems to the saturated orthogonal limit is also relatively small (Δ*J*_0_ ≈ +1.0 meV, see Figure 3c and Table 1). For DFB and DFN, their similarly higher planarity when relaxed and their moderate conformational responsiveness to out-of-plane E-fields are mirrored by a relatively higher magnetic responsiveness. Here, we observe lowering in AFM coupling strength of +2.6 meV for DFB and +2.4 meV for DFN relative to their relaxed values, for out-of-plane E-field strengths from 0–0.5 V/Å (see Figure 3c and Table 1). For the Ph linker, although the highly planar relaxed conformation has an associated high *J*, its relatively small out-of-plane conformational responsiveness leads to a correspondingly modest +1.1 meV decrease in the AFM coupling strength for an applied out-of-plane E-field strength of 0.5 V/Å.

For in-plane E-fields, the high conformational responsiveness of the ANA linker leads to a relatively large maximum increase in AFM coupling relative to that of its relaxed state (namely, from −0.9 to −3.9 meV; see Figure 3c and Table 1). Following the order of in-plane E-field conformational responsiveness for the dipolar linkers, the magnetic responsiveness is then progressively lower for DFA, DFN, and DFB. We note that for the most planar dipolar DFB linker, *J* reaches its most negative value for an in-plane E-field strength of 0.35 V/Å and then slightly increases. This nonlinear response is unlike the linear monotonic decrease in Δθ_0_ observed for DFB for the whole range of applied in-plane E-fields (see Figure 3b). In the case of the highly planar Ph linker, we seem to observe a more extreme version of this type of response, where all applied in-plane E-fields result in an increase in *J* (i.e., reducing the AFM coupling). As both Ph and DFB have planar relaxed conformations that are relatively resistant to further planarization (especially for Ph), the observed reduction in AFM coupling with applied E-field is likely to be mainly due to polarization-induced effects.

We note that *J* values for the projected fully coplanar conformations range between −21.1 meV (−167 cm^–1^) for DFB and −25.4 meV (−205 cm^–1^) for DFA (see the *J* values for θ = 0 in Figure 2b). These values are comparable with those derived for directly coupled nanographenes, for which a near planar conformation was obtained by mechanical force.^31^ As we see below, obtaining similar highly planar conformations for our dipolar linkers would require the application of very large E-fields.

In Table 1, we summarize the maximal Δθ_0_ and Δ*J*_0_ responses for all linkers for E-field strengths of 0.5 V/Å in each direction. From these data, we can assess the performance of each linker with respect to different application scenarios. For obtaining the highest AFM coupling strength, linkers that allow for higher π-orbital overlap are favored. In this sense, DFB is optimal among our considered dipolar linkers. DFB has a relatively high relaxed AFM coupling *J*_0_ = −7.7 meV, which can be further increased upon application of an in-plane E-field (*J* = −8.3 meV, Δ*J*_0_ = −0.6 meV). In terms of responsiveness, DFB also exhibits the largest decrease in AFM coupling (Δ*J*_0_ = +2.6 meV) with respect to out-of-plane *E*-fields. We also observe that it achieves this with the smallest Δθ_0_ response to the out-of-plane *E*-field, giving a Δ*J*_0_/Δθ_0_ performance of 0.24 meV/degree. We note that the extent of the conformational tunability of *J* achieved by DFB in our all-organic diradical system is significantly larger than that predicted for a DFB-bridged 2D metal–organic network.^58^ An in-plane E-field is better exploited by the ANA linker, in which the relatively low AFM coupling in the relaxed conformation (−0.9 meV) can be significantly increased (*J* = −3.9 meV, Δ*J*_0_ = −3.0 meV). This change represents a 433% increase in AFM coupling, with respect to the zero-field relaxed system. The ANA diradical system also shows highest cumulative total change in *J* (Δ*J*_tot_ = 3.9 meV) upon sequential application of out-of-plane and in-plane *E*-fields. These described changes in *J* would also result into significant changes in magnetic susceptibilities in ensembles of the respective diradicals (see the SI). We note that many of our *J* and Δ*J* values are significantly higher than those measured in phenyl-linked triangulenes in on-surface experiments (*J* = −2 meV).^59^ As such, the predicted magnetoelectric response of these molecular systems should be experimentally verifiable.

### Conformational Energy Profiles

A deeper understanding of the mechanical response of these systems is provided by examining the conformational energy profiles upon linker rotation (see Figure 4). In the absence of an applied E-field, such conformational energetic profiles can be understood from the interplay between steric hindrance and π-conjugation.^38^ Steric hindrance is produced by energetically unfavorable repulsive interactions between a linker and the TOT radicals, which are minimized in the orthogonal limit and increase with more planar conformations. As noted above, the degree of steric hindrance of the linkers follows the trend: Ph < DFB < DFN < DFA < ANA. The energetically favorable π-conjugation between a linker and the TOT radicals also increases with increased planarity and tends to increase with the size of the conjugated skeleton of the linker. The zero-field conformational energetic profiles are symmetric with respect to the orthogonal conformation (i.e., 90 °)—^38^ see black curves in Figure 4. These profiles for the smaller linkers (i.e., Ph, DFB, and DFN) show two clear minima corresponding to their respective, relatively low θ_0_ values (≤56°) separated by a maximum at θ = 90°. Here, rotating away from θ_0_ to smaller more planar conformations increases the steric hindrance and increases the system energy, whereas larger angles reduce the energetically favorable influence of π-conjugation. For the larger linkers (i.e., DFA and ANA), steric hindrance is more dominant, leading to higher, more orthogonal, θ_0_ values where π-conjugation is lower. Here, the energetically favorable increase in π-conjugation when twisting to more planar conformations is overcome by the large energy decrease due to steric hindrance. Likewise, when starting from a highly twisted θ_0_ value, the decrease in energetically favorable π-conjugation is very small. Overall, this tends to lead to a flatter low-energy region centered around θ = 90°. We note that these flatter regions tend to allow for easier linker rotation.

**Figure 4:**
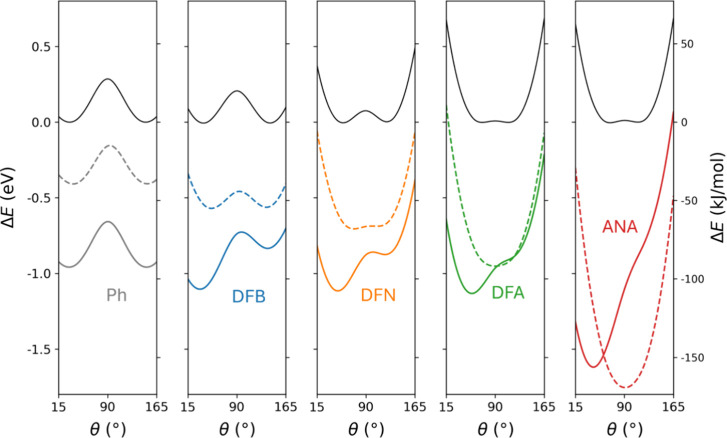
Conformational (i.e., twist angle) energy profiles for the five considered linkers. In all cases, the minimum energy of the profiles without applied E-field (black curves) are set to zero. Dashed (solid) colored profiles are obtained using an out-of-plane (in-plane) applied E-field of 0.5 V/Å.

The zero-field conformational profiles are modified after applying an external E-field (see colored curves in Figure 4). In all cases, the interaction of an applied E-field with the dipolar linkers acts to energetically stabilize all conformational profiles. The magnitude of this shift follows the magnitude of the fixed dipole moments of the linkers (i.e., Ph < DFB < DFN < DFA < ANA). Of this set, the relatively large dipole moment of ANA (8.17 D) compared to those of the other linkers (<3.0 D) contributes to a particularly large stabilization. We note that this fixed dipole perspective does not consider higher-order effects (e.g., polarization), which are also important in understanding the E-field conformational response of molecular systems.^38^ In the case of the nondipolar Ph linker, for example, the energetic stabilization of the zero-field profile for both in-plane and out-of-plane applied E-fields is effectively constant for all values of θ and due to polarization.

With the application of an E-field, the general energy-lowering effect on the profiles of the dipolar systems is more pronounced as the dipoles of the linkers become more aligned with the direction of the field (see Figure 4). For the DFB and DFN linkers, an out-of-plane E-field mainly results in a flattening of the central energy maximum centered at θ = 90°, with a corresponding slight positive shift in θ_0_ (see Table 1). For the DFA and ANA linkers, an out-of-plane field creates an energy minimum close to θ = 90° and a corresponding shift of θ_0_ from 69 to 89°. In contrast, an applied in-plane E-field favors molecular conformations that are closer to θ = 0°. However, as we approach planarity, steric hindrance begins to dominate the energy profiles in all cases. As such, for all systems, the in-plane E-field reinforces an energy minimum as close to θ = 0° as the respective steric hindrance permits. The most planar situation is achieved for the smaller DFB linker, for which the corresponding energy profile has a minimum at θ = 39°. This asymmetric E-field-induced effect also leads to a reduction of the barrier between the two zero-field minima. In the cases of DFA and ANA, the barrier is completely removed, which means that conformational twisting to the lower angle minimum would be spontaneous. For DFB and DFN, although a barrier between the two original minima remains, the small height of the barrier is such that the E-field is expected to bias the conformational twisting of the linkers to the lower-energy minimum. In such cases, upon removal of the E-field, the full barrier is recovered, which would tend to induce trapping of the linkers in the lower angle minimum. In the case of an ensemble of such diradicals (e.g., molecular crystals, network materials), this would lead to a ferroelectric ordering.

In Figure 5, we compare how linker conformation is related to the resultant *J* values with and without applied E-fields. Here, the colored curves show the *J* values in each system with respect to E-field-induced conformational twist angles below and above θ_0_ using in-plane or out-of-plane E-fields up to a maximum of 0.5 V/Å (see also Figure 3b,c). The black lines show the corresponding *J* values without applied E-fields when the linkers are constrained to be at the same range of conformational twist angles reached upon application of the E-field. For applied out-of-plane E-fields, the induced conformations (i.e., for θ > θ_0_) of each linker yield very similar *J* values to those of the respective conformationally constrained systems. This shows that out-of-plane E-fields only act to mechanically twist the linkers and do not significantly affect the resultant magnetic coupling for this range of angles. However, for in-plane E-fields, the resultant *J* values for increasingly planar conformations of the linkers (θ < θ_0_) increasingly diverge away (becoming less negative) from those *J* calculated for the same systems with no applied E-field. This divergence shows that in-plane fields are detrimental to the magnetic coupling strength for more planar linker conformations (i.e., for θ < θ_0_). As noted above, this situation is extreme for the most planar nondipolar Ph linker where all in-plane E-fields reduce the magnetic coupling strength. For the dipolar linkers with an applied in-plane E-field of 0.5 V/Å, the decrease in magnetic coupling strength for the E-field-induced case compared to the constrained zero-field case is found to range between 0.5 and 1.5 meV. Considering that Δ*J*_0_ values for this situation (i.e., with applied E-field) range between −0.6 and −3.0 meV (see Table 1), clearly this is a significant effect. It is likely that this effect is due to in-plane E-fields affecting the degree of the in-plane π-conjugation between the radical spin centers (e.g., via polarization of the linkers), which, thus, disrupts the main conduit for magnetic coupling in these systems.

**Figure 5:**
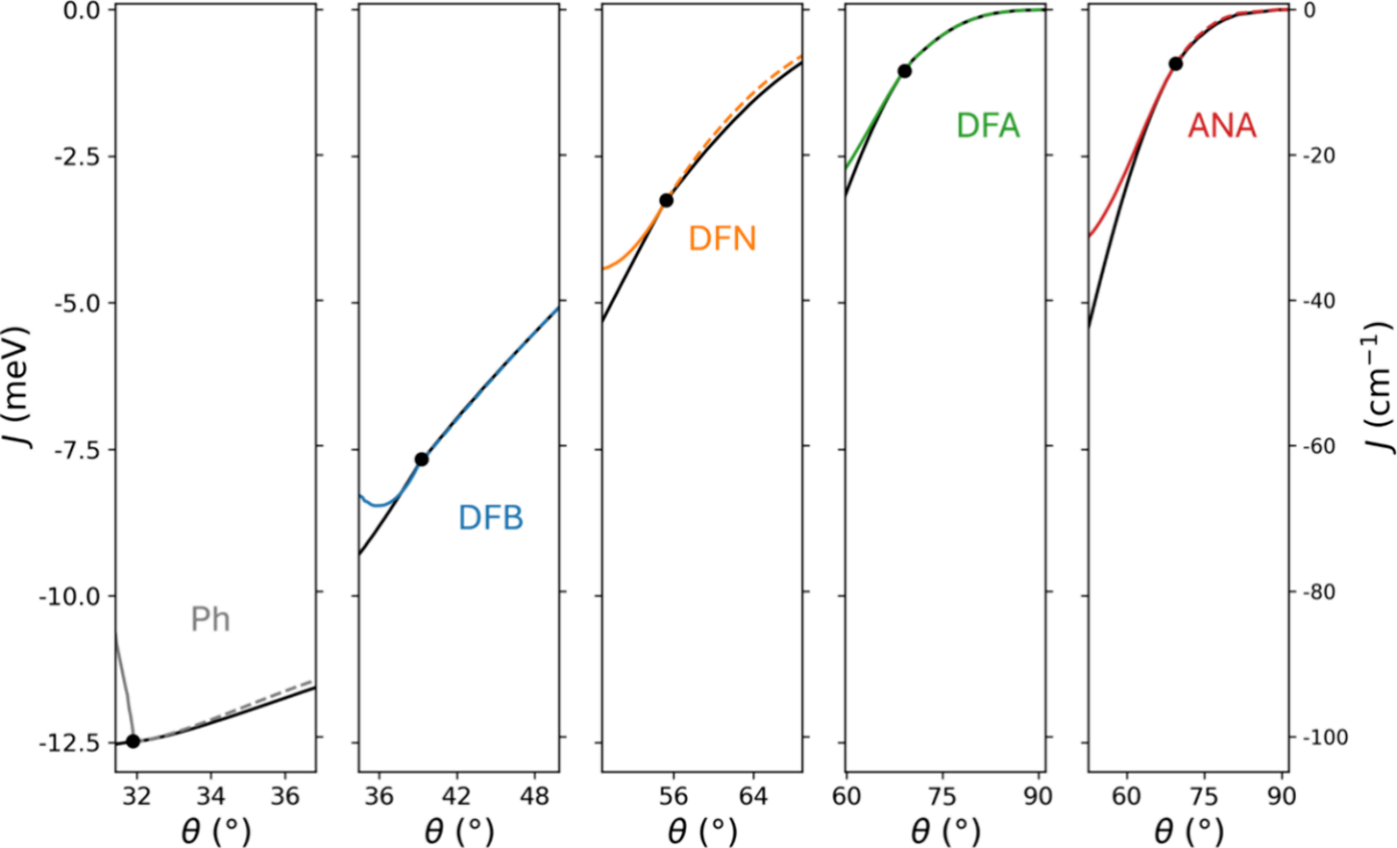
Comparison of *J* couplings with respect to the linker twist angle for the five considered linkers. The black data point in each case denotes θ_0_. The black line corresponds to single-point energy calculations for constrained linker conformations (reached upon application of the E-field) with no applied E-field. The colored lines correspond to conformations induced via applied in-plane E-fields (for θ < θ_0_, solid line) or out-of-plane E-fields (for θ > θ_0_, dashed line). The range of θ in each case corresponds to the maximum and minimum values obtained by applied E-fields with strengths of up to 0.5 V/Å (see Table 1 for Δθ_0_).

### From Diradicals to 2D Magnetoelectric Materials

To test whether the magnetoelectric response observed in our considered molecular systems could be employed in an extended 2D material, we used the highly responsive ANA-based diradical as a building block to construct a π-conjugated 2D covalent organic framework (2D-COF). Such 2D materials have great potential for incorporating a range of functionalities for device applications.^60–62^ Following theoretically predicted^35,63,64^ and experimentally realized^65–67^ examples of radical-based 2D-COFs, we build a honeycomb lattice-based ANA-2D-COF material (see Figure 6a) based on the ANA-based diradical.

**Figure 6:**
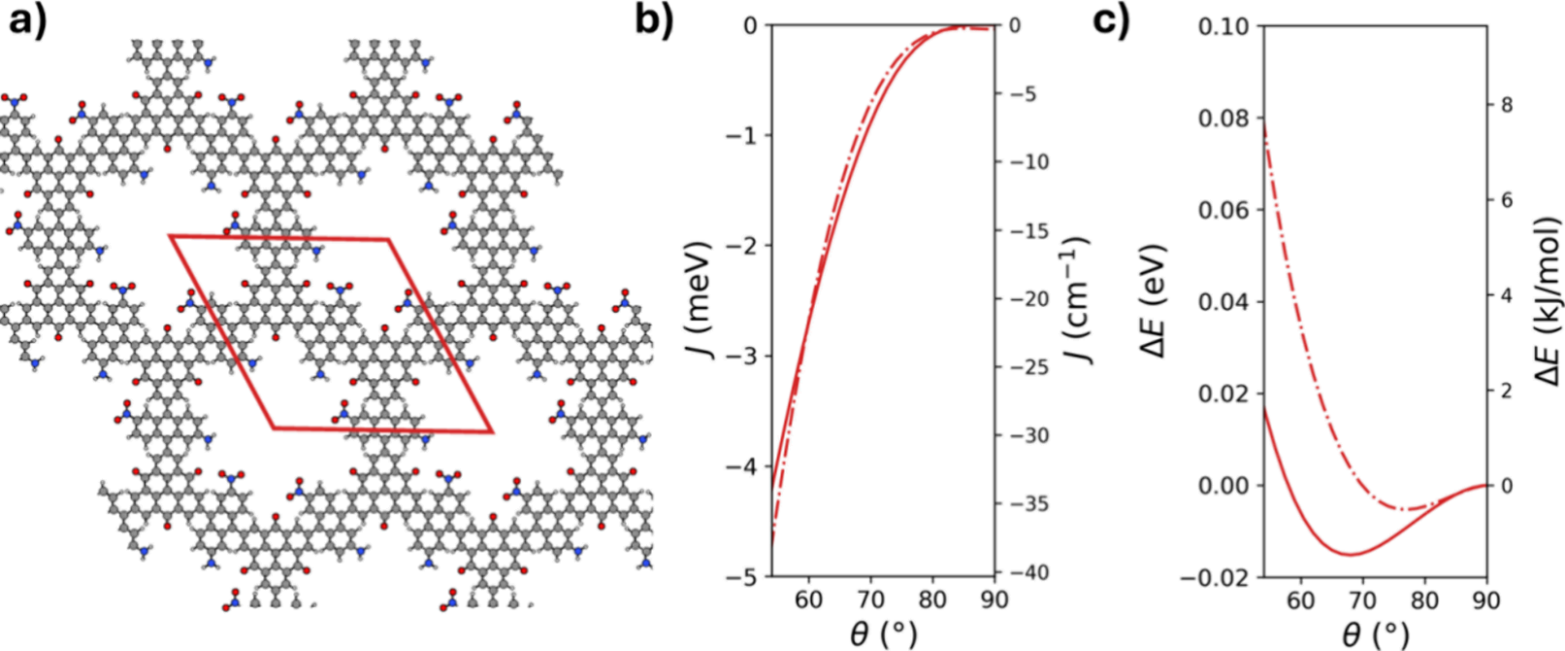
Comparison of the ANA-2D-COF and ANA-linked diradicals. (a) Structure of the ANA-2D-COF with the unit cell highlighted in red (the ANA linkers are shown in-plane to help visualize the structure). (b) Conformational magnetic coupling profiles and (c) conformational energy profiles, for both systems. Solid and dashed lines correspond to the ANA-based diradical and the ANA-2D-COF, respectively.

The ANA-2D-COF material takes advantage of the planar trigonal connectivity of the TOT units and the uniaxial ANA linkers (Figure 1). In Figure 6b, we compare the *J* values in the ANA-2D-COF (for AFM couplings between each pair of TOT radicals) with those in the ANA-linked diradical, with respect to the conformational twist angle. In the ANA-2D-COF, all linkers are twisted in the material simultaneously over the same range of E-field-accessible angles as in the corresponding diradical (i.e., 53° < θ < 90°—see Table 1). We find that the ANA-2D-COF exhibits slightly lower AFM couplings than the corresponding diradical when the linkers are twisted more out-of-plane (approximately θ > 60°). However, for more planar linker conformations, the ANA-2D-COF shows slightly stronger AFM couplings than the diradical. This latter effect is likely due to the increased π-conjugation resulting from long-range charge delocalization in the periodic ANA-2D-COF with respect to that in the molecular ANA-based radical.

In Figure 6c, we compare the normalized conformational energy profile of ANA-2D-COF with that of the diradical. For θ > 75°, the difference in energy between the two profiles is <1 meV and thus here we expect that the E-field-induced linker twisting response in the ANA-2D-COF would be very similar to that in the ANA-linked diradical. For progressively more planar conformations of the ANA linkers, the conformational energies for the ANA-2D-COF become increasingly larger than those for the diradical. This divergence is likely linked to collective interactions between linkers (e.g., steric, electrostatic), which tend to increase as the ANA linkers become closer in more planar conformations, and which are inherently absent in the diradical. Using smaller linkers such as DFB or DFN would likely reduce the impact of such interactions and allowing for a more facile E-field-induced in-plane collective conformations. As such, the magnetoelectric responsiveness of 2D-COFs with smaller dipolar linkers is likely to be more similar to the corresponding diradicals than when using larger dipolar linkers.

Apart from magnetic coupling, small fluorinated rings have also been used/proposed as twistable dipolar linkers in the context of (anti)­ferroelectric materials.^28,29,42,57^ As noted above, considering the individual energy profiles for DFB and DFN (see Figure 4), an application of an E-field followed by its removal would induce in-plane ferroelectric ordering in the corresponding 2D-COFs. In Figure 7a (top), we show a ferroelectric conformation of the ANA-2D-COF in which all linkers are fully out of plane and with the dipoles of all linkers pointing in the same direction. Here, the net polarization is orthogonal to the plane of the ANA-2D-COF. Although out-of-plane linker conformations are relatively low in energy, it is perhaps unlikely that an as-synthesized ANA-2D-COF would have the out-of-plane dipole components of all its linkers aligned. As such, very high E-fields would likely be required to twist the linkers through the plane of the material to align their dipoles. Following the conformational profile in Figure 6, removal of the applied E-field when in such a state would lead to a small angular relaxation of the linkers, for which a ferroelectric response would be maintained. The collective response of dipolar linkers to an applied in-plane E-field can also lead to a ferroelectric response (see the example in Figure 7a, lower). Here, all linkers can align with an applied E-field without twisting linkers through the plane of the material, and thus, the response should be achievable with relatively lower E-field strengths. Unlike in the out-of-plane ferroelectric conformation, the hexagonally ordered structure of the ANA-2D-COF entails that the alignments of the linker dipoles with a uniform applied E-field are generally not perfectly in line (Figure 7a, lower). We note that in this case, there is no net out-of-plane dipole as the direction of the dipoles of the linkers alternates up and down with respect to the plane of the 2D material. These two examples of collectively twisted linkers also possess different electronic properties. Although, as expected, the ANA-2D-COF has a finite band gap,^63^ the twisting of the linkers from an out-of-plane conformation to θ = 53° leads to a 0.19 eV decrease in the band gap (see Figure 7b). This effect is mainly due to shifting and broadening of the valence band in the latter case, which can be rationalized by the increased π-conjugated when the linkers are more in-plane.^35,36,37^

**Figure 7:**
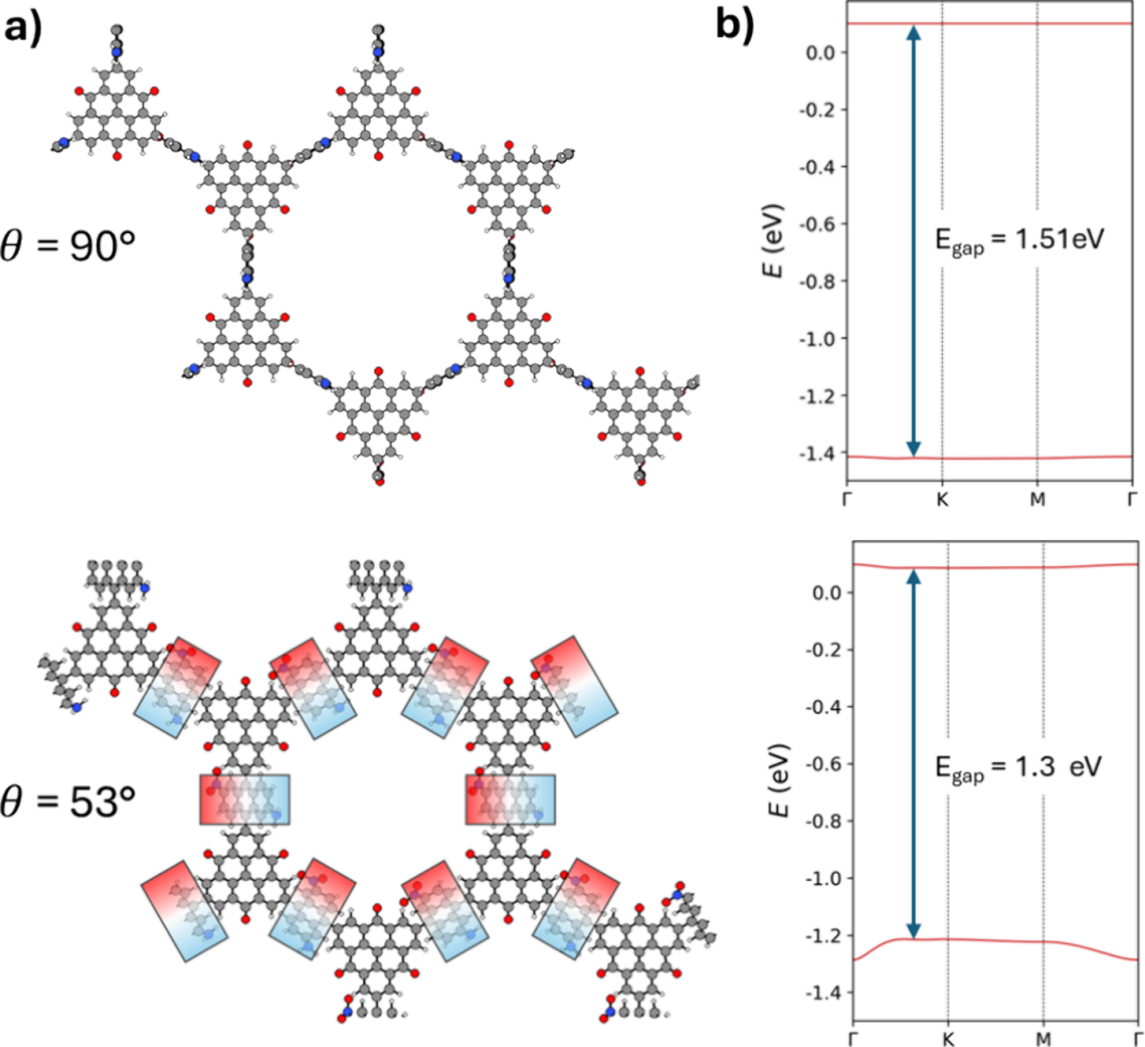
(a) Comparison of two ferroelectric ANA-2D-COF conformations for fully polarized out-of-plane ANA linkers (upper) and for θ = 53° (lower). In the latter case, we highlight the in-plane dipole ordering. (b) Corresponding valence and conduction bands for both ANA-2D-COF configurations with the minimum band gap (*E*_gap_) indicated.

## Conclusions

We have demonstrated the significant potential of a novel strategy to tune magnetic properties in all-organic systems with applied E-fields. This approach leverages twistable dipolar linkers as E-field-responsive molecular units that can control magnetic interactions in molecular diradicals and extended radical-based materials (e.g., 2D COFs). We show that experimentally realizable E-fields of moderate strengths of up to 0.5 V/Å induce conformational changes of dipolar linkers, which affects π-conjugation and thus modulates the exchange spin coupling, *J*, between radical centers. In the considered diradicals, measurable cumulative changes in *J* of up to 3.9 meV (31 cm^–1^) can be achieved upon applying in-plane and out-of-plane E-fields. Notably, in some cases, the E-field can induce switching between paramagnetic and AFM responses. In the corresponding extended 2D materials, the spin centers can exhibit a stronger AFM coupling, promising increased CME performance. However, here, the resistance to conformational twisting is also increased for the larger linkers. This suggests that the promising magnetoelectric response of the diradicals would be easier to reproduce in extended 2D-COFs using smaller dipolar linkers. We note that strain-induced changes in (FM) magnetic coupling (in inorganic 2D systems) of a similar magnitude to those we find have been proposed as being potentially useful for magnonics.^68^ We also note that our 2D COFs could also display E-field induced ferroelectric responses.

Overall, our proof-of-principle study lays a foundation for the rationally designing innovative 2D all-organic CME materials. Given the vast number of possibilities for molecular design when combining stable radicals and dipolar linkers, it is clear that there is much room for enhancing CME performance (i.e., larger changes in *J* with smaller E-field strengths). As such, our study paves the way for developing functional materials for potential future technological applications (e.g., magnonics, spintronics, quantum computing, sensing, ferroelectrics), with the inherent benefits of organic systems (e.g., flexibility, sustainability, low spin–orbit coupling).

## Supplementary Material



## Data Availability

A data set collection of computational results is available in the ioChem-BD repository^69^ and can be accessed via http://doi.org/10.19061/iochem-bd-6-531

## Notes

Geometrical constraints used in optimizations, calculated changes in magnetic susceptibilities in ensembles of the respective diradicals, conformational energy profiles for different applied E-fields, calculation of *J* using DFT/PBE0 and a def2-TZVPP basis set, comparison of *J* values calculated using DFT/PBE0 and NEVPT2, comparison of *J* using Yamaguchi versus Noodleman projections, comparison of vertical versus adiabatic *J*, and organization of associated openly accessible data files (PDF)
