# Supplementary material for: Rational Design of Electric Field-Responsive Building Blocks for All-Organic 2D Magnetoelectric Materials
Source: J Am Chem Soc. 2025 Jun 17;147(26):22550–61. doi: 10.1021/jacs.5c02910 (PMC12232323; doi:10.1021/jacs.5c02910)
Supplement: Supplementary file 1 [file ja5c02910_si_001.pdf]

Supporting Information

Rational Design of Electric Field Responsive Building Blocks for All-Organic 2D Magnetoelectric Materials

Kílian Jutglar-Lozano[1], Mercè Deumal[1], Jordi Ribas-Arino[1*], Stefan T. Bromley[1,2*]

[1]*Departament de Ciència de Materials i Química Física & Institut de Química Teòrica i Computacional (IQTCUB), Universitat de Barcelona, c/Martí i Franquès 1-11, 08028 Barcelona, Spain*

[2]*Institució Catalana de Recerca i Estudis Avançats (ICREA), Passeig Lluís Companys 23, 08010 Barcelona, Spain*

[*]Corresponding authors: j.ribas@ub.edu, s.bromley@ub.edu .

S1. Diradical constraints to mimic 2D materials

S2. Constraints to obtain conformational energy profiles

S3. Calculated changes in magnetic susceptibilities in ensembles of the respective diradicals

S4. Conformational energy profiles for different applied E-fields

S5. Calculation of *J* using DFT/PBE0 and a def2-TZVPP basis set

S6. Comparison of *J* values calculated using DFT/PBE0 and NEVPT2

S7. Comparison of *J* using Yamaguchi versus Noodleman projections

S8. Comparison of vertical versus adiabatic *J*

S9. Organization of data in the open access repository

S1. Diradical constraints to mimic 2D materials

To obtain diradical relaxed structures that reflect their role in 2D networks, geometric constraints must be applied to maintain coplanarity of the TOT radicals. These are established to avoid motions that would make the construction of their two-dimensional analogues incompatible by applying the periodic boundary conditions. The following considerations are the same for all the diradicals employed. The two main motions that need to be constrained are the linear bend of the diradical along its long axis, and the relative rotation between the TOT spin centres (see Figure S1).

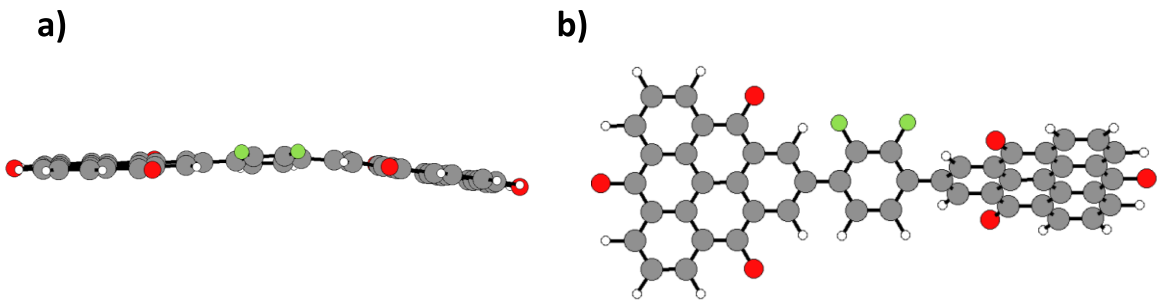

Fig. S1. a) Side view of the linear bending along the long axis of the DFB diradical. b) Top view of the relative twisting of the TOT radicals. Atom colour key: C – grey, H – white, O – red, F – green.

The linear bending can be avoided by fixing the angle formed by the blue marked atoms in Figure S2 to 180°, and the twisting can be avoided by fixing the dihedral angles defined in Figure S3 to 0°.

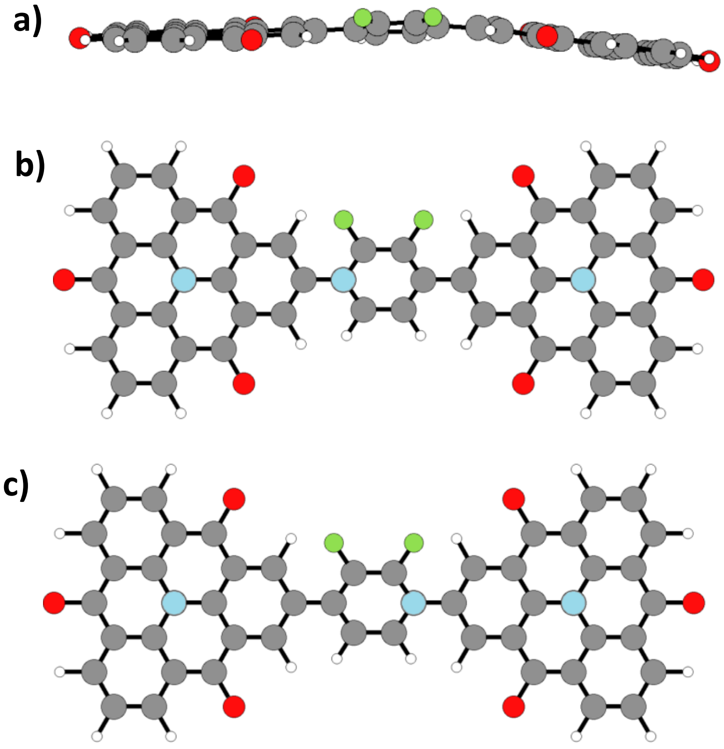

Fig. S2. a) Diradical linear bending along its long axis. This motion can be restricted by fixing the angles marked by the light blue coloured carbon atoms in b and c) to 180°.

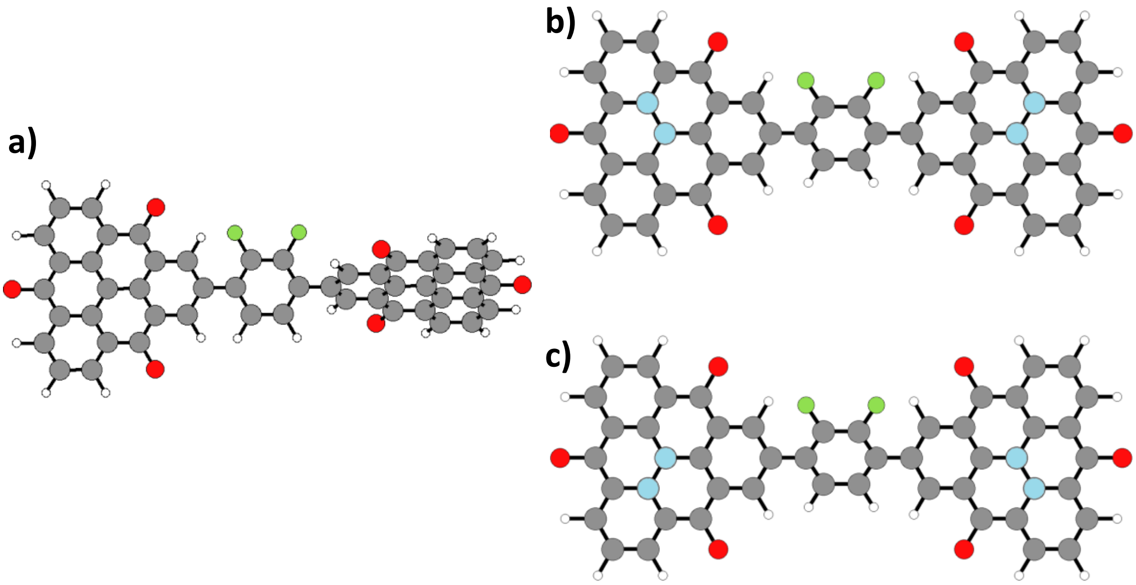

Fig. S3. a) Relative twisting of the TOT radical units. This motion is avoided by fixing the dihedral angles marked by the light blue coloured carbon atoms in b) and c) to 0°.

S2. Constraints to obtain conformational energy profiles

To perform the conformational studies, the structures are optimized at different conformations where the rotation of the linker is fixed. In the case of the diradicals, this is done by fixing the dihedral angle marked in Figure S4.

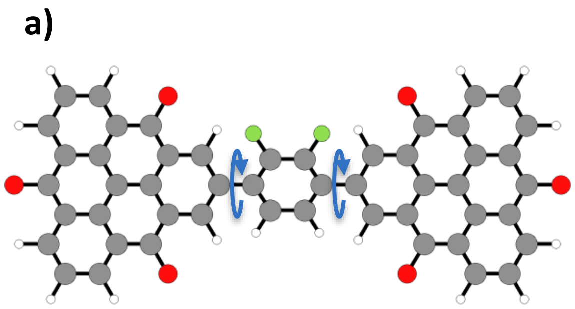

Fig. S4. a) Conformational energy profiles are obtained by rotating and fixing the linkers at different dihedral angles (see blue arrows indicating rotation axis). b) The dihedral angle of the linker defined by the light blue coloured carbon atoms is fixed to maintain the conformation in the diradicals.

In the case of the 2D-material, the strategy is the same, but because of the software employed (FHI-AIMS) does not allow freezing dihedral angles, this is done by freezing the *z* cartesian component of key atoms of the linker's skeleton.

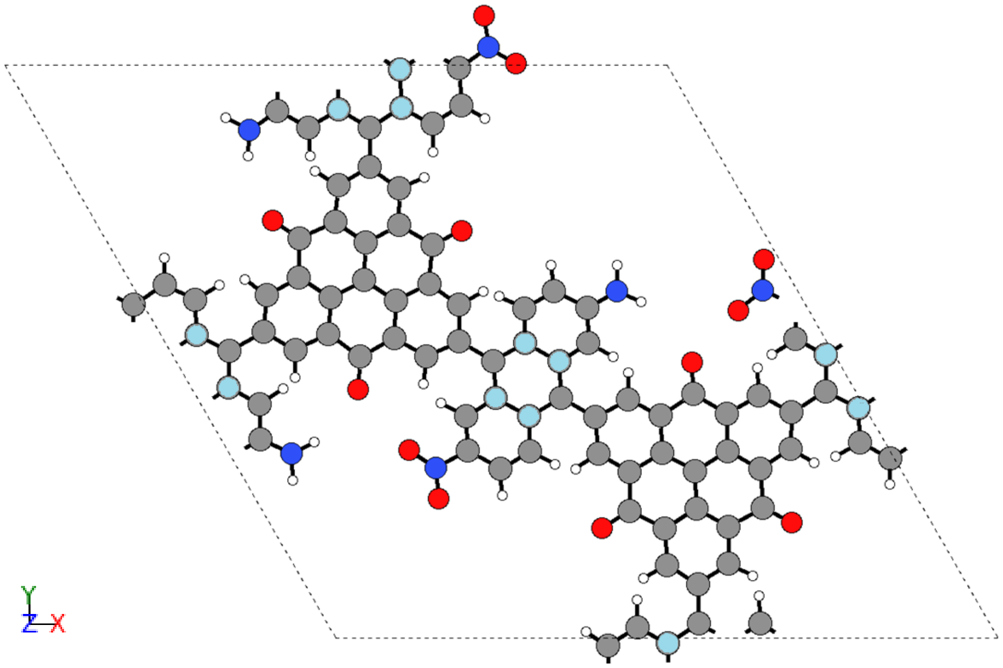

Fig. S5. Top view of the 2D-material using the ANA linker. The conformation of the linker is fixed by freezing the *z* cartesian component of the light blue coloured carbon atoms. Dark blue indicates N atoms.

S3. Calculated changes in magnetic susceptibilities in ensembles of the respective diradicals

Magnetic susceptibilities were computed as a function of J and temperature using Bleaney-Bowers equation[1] resulting from the Heisenberg-Dirac-Van Vleck Hamiltonian $\widehat{H} = -2J\hat{S}_1\hat{S}_2$ for $\hat{S}_1 = \hat{S}_2 = ½$:

$$\chi_m T = \frac{N_A {\mu_B}^2 g^2}{k} \frac{2e^{\frac{2J}{kT}}}{1 + 3e^{\frac{2J}{kT}}}$$

The reported calculated χ_mT magnetic susceptibility values are consistent with the achievement of AFM coupling, since χ_mT is always lower than 0.75, which is the value for two totally decoupled ½ spins (see Figure S6). Moreover, the variation of the observed magnetic susceptibilities (±0.05 emu K mol^{-1} to ±0.25 emu K mol^{-1}) obtained can be measured under experimental conditions[2]. The maximum responses are summarised in table S1.

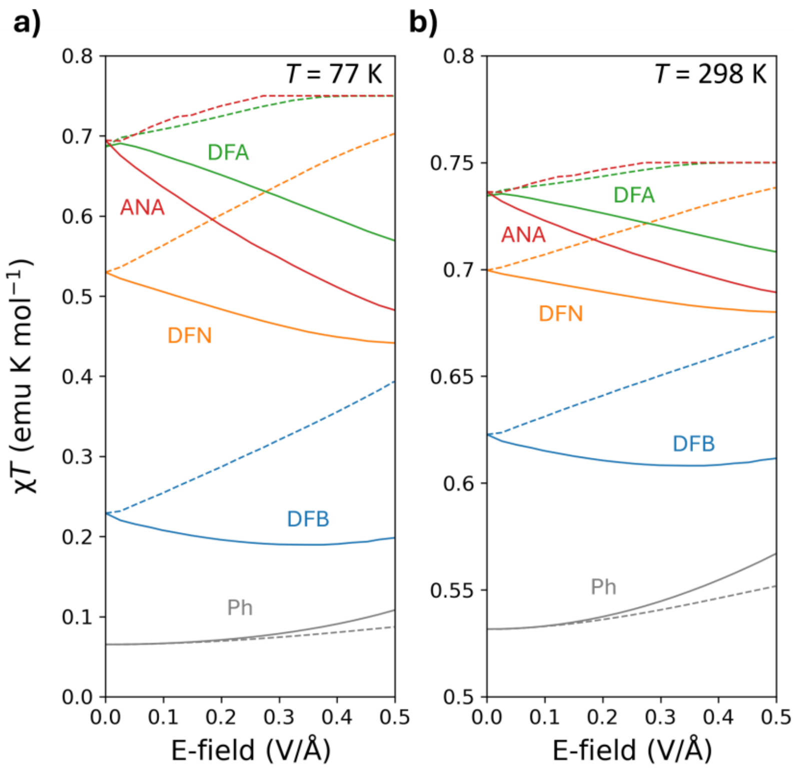

Fig. S6. Variation of calculated magnetic susceptibility (χ_mT) with respect to applied E-field strength. In-plane and out-of-plane E-fields are indicated by solid and dashed lines, respectively. The E-field strength in each case ranges between 0 and 0.5 V/Å in steps of 0.025 V/ Å.

Table S1. Summary of the maximum conformational and magnetic E-field response of each considered linker with respect to the corresponding relaxed system with no applied E-field at 77 K. Columns 2 and 3 show the relaxed values of θ (θ_0) and $\chi_m T$ ($\chi_m T_0$) for each linker. Columns 4 (6) and 5 (7) show the maximum changes in θ ($\Delta\theta_0$) and $\chi_m T$ ($\Delta\chi_m T_0$) with respect the respective relaxed values upon application of a 0.5 V/Å in-plane (out-of-plane) E-field. Columns 8 and 9 show the cumulative total changes of θ ($\Delta\theta_{tot}$) and $\chi_m T$ ($\Delta\chi_m T_{tot}$) (i.e. sum of absolute changes) for both applied E-field directions.

System	Relaxed		In-plane E-field		Out-of-plane E-field		Total response	
	$\boldsymbol{\theta_0}$ #	$\boldsymbol{\chi_m T_0}$ §	$\boldsymbol{\Delta\theta_0}$ #	$\boldsymbol{\Delta\chi_m T_0}$ §	$\boldsymbol{\Delta\theta_0}$ #	$\boldsymbol{\Delta\chi_m T_0}$ §	$\boldsymbol{\Delta\theta_{tot}}$ #	$\boldsymbol{\Delta\chi_m T_{tot}}$ §
**Ph**	32	0.07	-1	+0.04	+5	+0.02	6	0.02
**DFB**	39	0.23	-5	-0.03	+11	+0.16	16	0.20
**DFN**	55	0.53	-6	-0.09	+14	+0.17	20	0.26
**DFA**	69	0.69	-9	-0.12	+22	+0.06	31	0.18
**ANA**	69	0.69	-16	-0.21	+22	+0.06	38	0.27

All dihedral twist angles θ are given in degrees (°)

§ All magnetic susceptibilities $\boldsymbol{\chi_m T}$ are given in emu K mol^{-1}.

S4. Conformational energy profiles for different applied E-fields

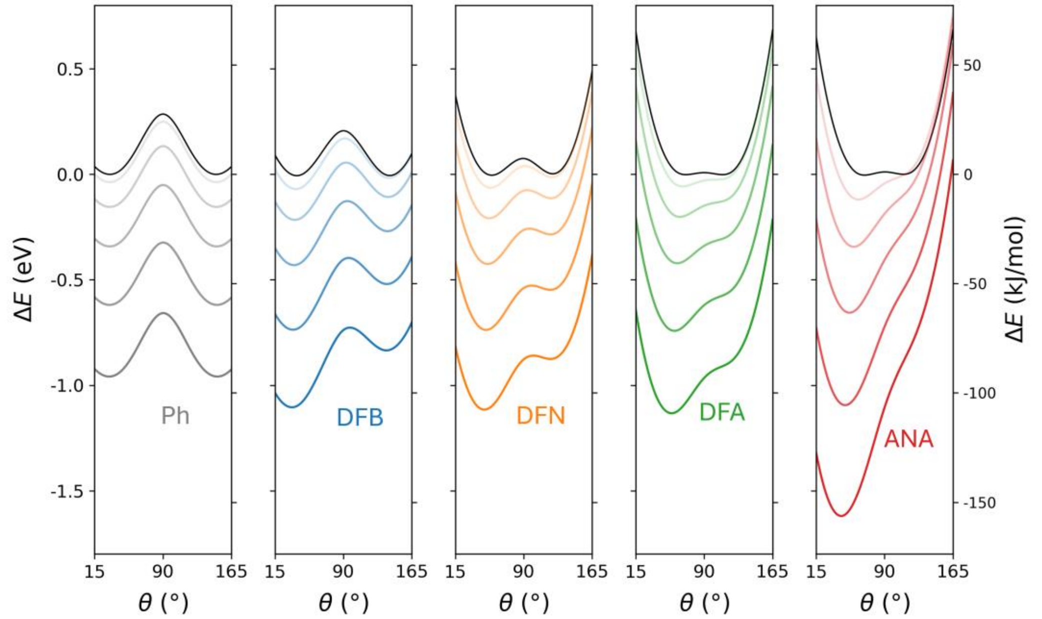

Fig. S7. Conformational (i.e., twist angle) energy profiles for the five considered linkers. In all cases the minimum energy of the profiles without applied E-field (black curves) are set to zero. Solid coloured profiles are obtained using in-plane applied E-fields of 0.1 V/Å, 0.2 V/Å, 0.3 V/Å, 0.4 V/Å and 0.5 V/Å. The E-field strength is denoted with the increasing intensity of the coloured lines.

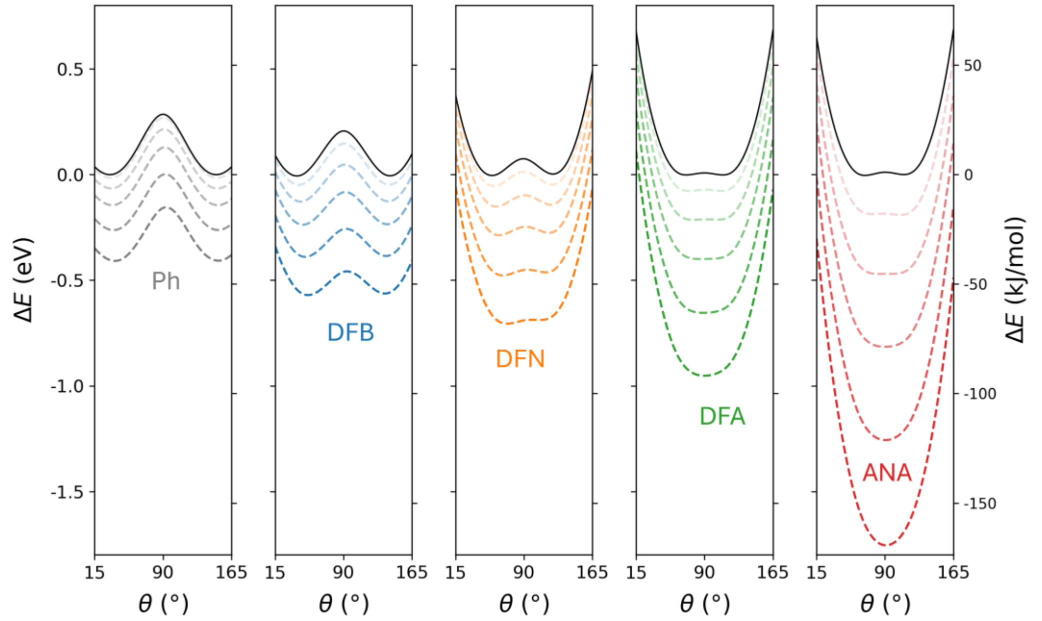

Fig. S8. Conformational (i.e., twist angle) energy profiles for the five considered linkers. In all cases the minimum energy of the profiles without applied E-field (black curves) are set to zero. Dashed coloured profiles are obtained using out-of-plane applied E-fields of 0.1 V/Å, 0.2 V/Å, 0.3 V/Å, 0.4 V/Å and 0.5 V/Å. The E-field strength is denoted with the increasing intensity of the coloured lines.

S5. Calculation of J using DFT/PBE0 and a def2-TZVPP basis set

The effect of the basis set on the values of J obtained for DFB and the DFN diradicals at the PBE0 level were assessed by carrying out single-point calculations using the def2-TZVPP basis set[3] for three different conformations: 1) relaxed without E-field, 2) when applying the maximum in-plane E-field strength and 3) when applying the maximum out-of-plane E-field strength. The results collected in the table below show minor differences between the two basis sets.

Table S2. Comparison of the J values obtained at the PBE0/6-311g(d,p) and PBE0/def2-TZVPP levels. All values of J are given in cm^{-1}.

System	Relaxed		In-plane 0.5 V/Å		Out-of-plane 0.5 V/Å	
DFB	$\theta = 39°$		$\theta = 34°$		$\theta = 50°$	
	6-311 g(d,p)	def2-TZVPP	6-311g(d,p)	def2-TZVPP	6-311 g(d,p)	def2-TZVPP
	-61.9	-62.7	-66.8	-67.5	-40.9	-41.5
DFN	$\theta = 55°$		$\theta = 49°$		$\theta = 69°$	
	6-311 g(d,p)	def2-TZVPP	6-311 g(d,p)	def2-TZVPP	6-311 g(d,p)	def2-TZVPP
	-26.2	-26.3	-35.7	-35.9	-6.4	-6.4

S6. Comparison of J values calculated using DFT/PBE0 and NEVPT2

The values of J obtained for DFB at the PBE0 level were assessed against CASSCF(12,12)/NEVPT2 calculations for three different conformations: 1) relaxed without E-field, 2) when applying the maximum in-plane E-field strength and 3) when applying the maximum out-of-plane E-field strength. For each conformation, the NEVPT2 calculation was carried out on top of a state-average CASSCF wave function (orbitals optimized for the average of the lowest-energy singlet and lowest-energy triplet states), using a def2-TZVP(-f) basis set, as implemented in the ORCA 6.0 program.[4]

Table S3. Comparison of the J values obtained at the CASSCF(12,12)/NEVPT2 level with those obtained at the PBE0 level for the DFB diradical. All values of J are given in cm^{-1}.

**Relaxed**		**In-plane 0.5 V/Å**[a]		**Out-of-plane 0.5 V/Å**[a]	
$\boldsymbol{\theta = 39°}$		$\theta = 34°$		$\theta = 50°$	
**NEVPT2**	PBE0	NEVPT2	PBE0	NEVPT2	PBE0
**-60.2**	-61.9	-71.0	-75.3	-39.1	-41.0

[a] These values are obtained without including the E-field in the single point calculations.

S7. Comparison of J using Yamaguchi versus Noodleman projections

The values of J obtained with the Noodleman projection scheme were assessed against the values obtained using the Yamaguchi formula[5] for three different conformations: 1) relaxed without E-field, 2) when applying the maximum in-plane E-field strength and 3) when applying the maximum out-of-plane E-field strength. The results in the table below show minor differences using either approach to obtain J.

Table S4. Comparison of the J values obtained using Yamaguchi's formula with those obtained through the Noodleman's projection scheme. All values of J are given in cm^{-1}.

System	Relaxed		In-plane 0.5 V/Å		Out-of-plane 0.5 V/Å	
	Noodleman	Yamaguchi	Noodleman	Yamaguchi	Noodleman	Yamaguchi
**Ph**	-100.6	-100.3	-85.9	-85.6	-92.3	-91.9
**DFB**	-61.9	-61.8	-66.8	-66.6	-41.0	-40.9
**DFN**	-26.2	-26.2	-35.7	-35.7	-6.4	-6.4
**DFA**	-8.4	-8.4	-21.9	-21.9	0.0	0.0
**ANA**	-7.4	-7.4	-31.2	-31.2	0.0	0.0

S8. Comparison of vertical versus adiabatic *J*

The optimized geometries of the triplet state are very similar to those of the singlet state for all the diradicals as demonstrated in the table below. This is assessed for three different conformations: 1) relaxed without E-field, 2) when applying the maximum in-plane E-field strength and 3) when applying the maximum out-of-plane E-field strength.

Table S5. RMSD (Root Mean Squared Difference) values between the optimized geometries of the diradicals in the singlet and triplet states. All values are given in Å.

System	Relaxed	In-plane 0.5 V/Å	Out-of-plane 0.5 V/Å
**Ph**	0.014	0.011	0.013
**DFB**	0.008	0.007	0.007
**DFN**	0.010	0.008	0.008
**DFA**	0.011	0.008	0.000
**ANA**	0.013	0.007	0.000

As a result of the small RMSDs, the difference between vertical and adiabatic *J* is very small, especially for the dipolar linkers.

Table S6. Vertical and adiabatic J values for all the systems. All values are given in cm^{-1}.

System	Relaxed		In-plane 0.5 V/Å		Out-of-plane 0.5 V/Å	
	Vertical	Adiabatic	Vertical	Adiabatic	Vertical	Adiabatic
**Ph**	-100.6	-94.9	-85.9	-81.7	-92.3	-87.0
**DFB**	-61.9	-59.6	-66.8	-64.7	-41.0	-39.4
**DFN**	-26.2	-25.0	-35.7	-34.5	-6.4	-6.1
**DFA**	-8.4	-8.0	-21.9	-21.0	0.0	0.0
**ANA**	-7.4	-7.0	-31.2	-30.2	0.0	0.0

S9. Organization of data in the open access repository

All calculations performed have been uploaded to the publicly accessible and free repository ioChem-BD (https://doi.org/10.19061/iochem-bd-6-445). For each calculation, the corresponding input file and a summary of the key results extracted from the output are provided. The calculations are systematically organized to ensure easy access to the relevant information. Figure S9 presents a schematic representation of this organizational structure n the case of the 2D-material, the strategy is the same, but because of the software employed (FHI-AIMS) does not allow freezing dihedral angles, this is done by freezing the *z* cartesian component of key atoms of the linker's skeleton.

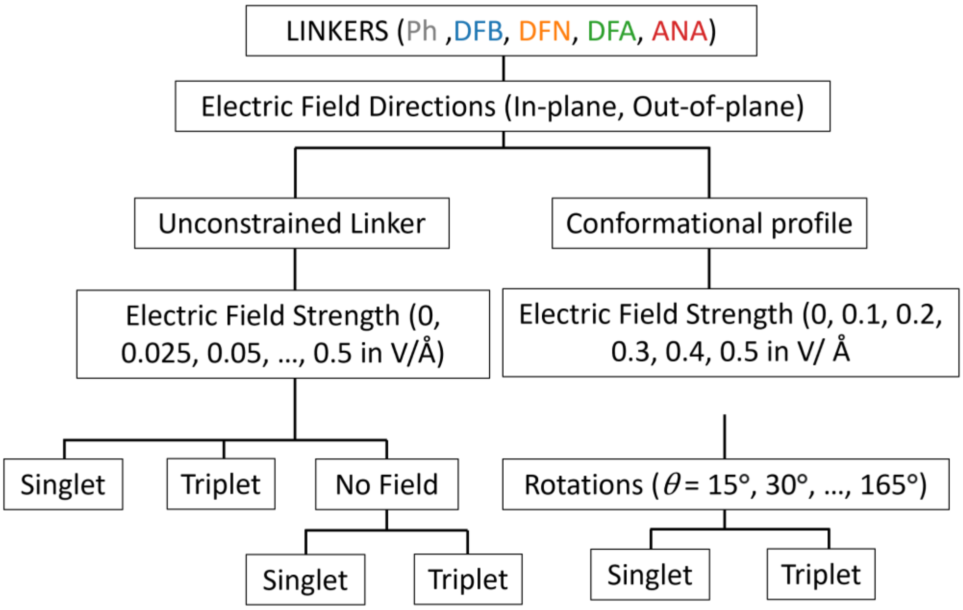

Fig. S9. Schematic representation of the computational dataset organization. The calculations are categorized based on the type of linker (Ph, DFB, DFN, DFA, ANA), the presence or absence of an external E-field, and the specific computational conditions applied. When an electric field is present, calculations explore different E-field directions (in-plane and out-of-plane) and strengths. Special consideration is given to structures containing the ANA linker, where its 2D materials coordinates are included in the conformational profile without E-field applied. DOI: https://doi.org/org/10.19061/iochem-bd-6-531.

References

[1] Bleaney, B.; Bowers, K. D. Anomalous paramagnetism of copper acetate. *Proc. R. Soc. Lond. Ser. A* **1952**, *214*, 451-465. https://doi.org/10.1098/rspa.1952.0181

[2] Umezono, Y.; Fujita, W.; Awaga, K. Coordination Bond Formation at Charge-Transfer Phase Transition in $(BDTA)_2[Co(mnt)_2]$. *J. Am. Chem. Soc.* **2006**, *128*, 1084-1085. https://doi.org/10.1021/ja057207i

[3] Weigend, F.; Ahlrichs, R. Balanced basis sets of split valence, triple zeta valence and quadruple zeta valence quality for H to Rn: Design and assessment of accuracy. *Phys. Chem. Chem. Phys*. **2005**, *7*, 3297–3305. https://doi.org/10.1039/B508541A

[4] Neese, F. Software Update: The ORCA Program System—Version 6.0. WIREs Computational Molecular Science, 2025. https://doi.org/10.1002/wcms.70019.

[5] Yamaguchi, K.; Jensen, F.; Dorigo, A.; Houk, K. N. A Spin Correction Procedure for Unrestricted Hartree-Fock and Møller-Plesset Wavefunctions for Singlet Diradicals and Polyradicals. Chemical Physics Letters, 1988, 149, 537–542. https://doi.org/10.1016/0009-2614(88)80378-6.
